# Inferring Metabolic States in Uncharacterized Environments Using Gene-Expression Measurements

**DOI:** 10.1371/journal.pcbi.1002988

**Published:** 2013-03-21

**Authors:** Sergio Rossell, Martijn A. Huynen, Richard A. Notebaart

**Affiliations:** 1 Department of Bioinformatics (CMBI), Centre for Molecular Life Sciences, Radboud University Nijmegen, The Netherlands; 2 Centre for Systems Biology and Bioenergetics (CSBB), Radboud University Nijmegen, The Netherlands; The Centre for Research and Technology, Hellas, Greece

## Abstract

The large size of metabolic networks entails an overwhelming multiplicity in the possible steady-state flux distributions that are compatible with stoichiometric constraints. This space of possibilities is largest in the frequent situation where the nutrients available to the cells are unknown. These two factors: network size and lack of knowledge of nutrient availability, challenge the identification of the actual metabolic state of living cells among the myriad possibilities. Here we address this challenge by developing a method that integrates gene-expression measurements with genome-scale models of metabolism as a means of inferring metabolic states. Our method explores the space of alternative flux distributions that maximize the agreement between gene expression and metabolic fluxes, and thereby identifies reactions that are likely to be active in the culture from which the gene-expression measurements were taken. These active reactions are used to build environment-specific metabolic models and to predict actual metabolic states. We applied our method to model the metabolic states of *Saccharomyces cerevisiae* growing in rich media supplemented with either glucose or ethanol as the main energy source. The resulting models comprise about 50% of the reactions in the original model, and predict environment-specific essential genes with high sensitivity. By minimizing the sum of fluxes while forcing our predicted active reactions to carry flux, we predicted the metabolic states of these yeast cultures that are in large agreement with what is known about yeast physiology. Most notably, our method predicts the Crabtree effect in yeast cells growing in excess glucose, a long-known phenomenon that could not have been predicted by traditional constraint-based modeling approaches. Our method is of immediate practical relevance for medical and industrial applications, such as the identification of novel drug targets, and the development of biotechnological processes that use complex, largely uncharacterized media, such as biofuel production.

## Author Summary

Metabolic fluxes are steady-state rates of metabolite interconversion within living cells. They determine the rates of growth and product formation, and are of biotechnological and medical importance. An important and pressing question is how to identify the actual distribution of fluxes in living cells among the manifold possibilities that complex metabolic networks allow. One way to address this question is to constrain the space of possibilities using gene-expression measurements. Here we present a method that uses gene-expression measurements to infer the metabolic state of cells growing in uncharacterized environments. We applied this method to model the metabolism of *Saccharomyces cerevisiae* grown with glucose or ethanol as main energy source. Our modeling enables the prediction of genes that are essential for growth in either environment. We also show that our method predicts aspects of the energy metabolism of these cultures that are in large agreement with what is known about yeast physiology. Our method is of direct practical importance in the fields of biotechnology and medicine, such as *in vivo* drug target identification, where nutrient conditions are largely unknown.

## Introduction

The metabolic state of a cell is defined by the distribution of all its metabolic fluxes. It constitutes a significant aspect of cellular phenotype and is of both medical and industrial relevance. There are, for instance, striking differences in the metabolic states of healthy and cancer cells. Healthy cells rely predominantly on oxidative phosphorylation as a means to drive ATP synthesis, but cancer cells ferment profusely, converting the majority of the glucose and glutamine they consume into lactate [1]. Moreover, some cancer cell lines have been shown to synthesize lipids by reductive carboxylation of glutamine-derived α−ketoglutarate [2]. On the industrial side, efforts towards redirecting metabolic fluxes for the production of industrially relevant metabolites continue to drive the study of metabolism in industrial microorganisms [3].

**Figure 1:**
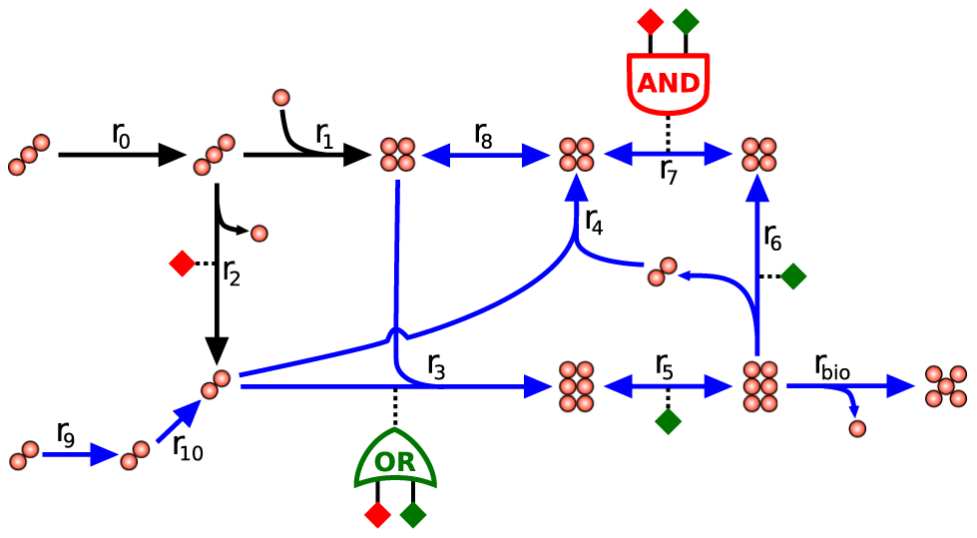
Toy metabolic network. This network enables the synthesis of a five-carbon biomass precursor from a two- or from a three-carbon substrate. The network's gene-to-reaction mapping is depicted with diamonds indicating highly (green) and lowly (red) expressed genes. Two reactions (r3 and r7) are each associated with two genes. For r3 any of the two gene-products suffices to catalyze the reaction (OR logic gate). For r7, both gene-products are needed together to bring about catalysis (AND logic gate). Highlighted in blue is a flux distribution that maximizes the number of reactions that are consistent with their gene expression. Note that the highlighted flux distribution results in four agreements between reaction fluxes and their expression (r2, r3, r5, and r6) and one disagreement (r7).

**Figure 2:**
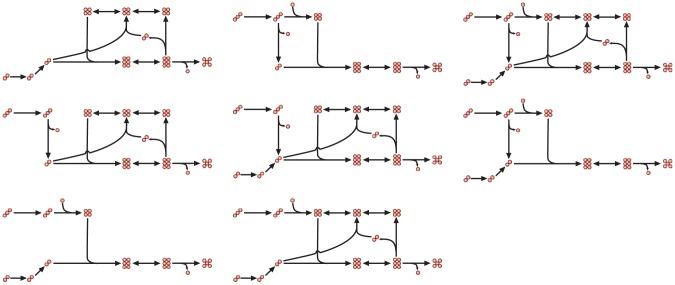
Possible flux distributions in the toy metabolic network. The toy metabolic network in Figure 1 supports eight different flux distributions, all of which are consistent with the model's stoichiometric and thermodynamic constraints.

The advent of genome-scale constraint-based models of metabolism enables the study of metabolic fluxes in whole-cells. The large size of these metabolic models, however, entails the challenge of identifying the actual metabolic state of cells among the myriad possibilities allowed by stoichiometric and thermodynamic constraints. To illustrate this point, Figure 1 shows a toy pathway in which a five-carbon biomass precursor is synthesized from a two- or from a three-carbon substrate. Figure 2 shows that this very small toy network supports no less than 8 different flux distributions. This multiplicity of possible flux distribution motivates studies aimed at the utilization of experimental data to reduce the flux distribution space and improve the predictions of metabolic states using constraint-based models of metabolism [4].

Nutrient availability places an enormous constraint on the space of possible flux distributions. For instance, in our toy model, there are only two flux distributions compatible with the absence of the two-carbon substrate (Figure 2). However, in many important medically and industrially relevant situations, cells live in poorly characterized environments. For example, the media used in industrial fermentations have complex and variable compositions [5], and the nutrients available to pathogens in host environments are poorly characterized [6]. The difficulties associated with the precise determination of the nutrients available to cells in complex variable media or within a host, stand in stark contrast with the relative ease with which transcript abundances can be quantified.

It is reasonable to expect gene expression to be a major determinant of the cell's flux distribution. However, the understanding of the relationship between transcript levels and metabolic fluxes has been challenged by the confounding observations that, for both prokaryotes and eukaryotes, transcript levels correlate poorly with protein concentrations [7]–[16]. Furthermore, for *Saccharomyces cerevisiae*, fluxes have been reported to correlate poorly with both transcripts and protein levels [17]–[19]. Despite these poor correlations, when transcript and protein measurements from *Escherichia coli* were analyzed in the light of a genome-scale model of metabolism, evidence for a coordinated expression of metabolic genes became apparent [20].

Several methods for integrating transcript measurements and genome-scale models of metabolism have been proposed (e.g. [21]–[24], reviewed in [25]). We highlight two methods that were proposed to build tissue- and condition-specific models using gene expression as their sole input [26], [27]. Importantly these methods do not require knowledge upon nutrient availability nor require assumptions about the metabolic objectives of cells. Jerby et al. [26] proposed the Model Building Algorithm (MBA). This method requires the definition of a set of ‘high-probability core reactions’ (cH) by, for instance, selecting reactions associated with highly expressed genes [28]. The MBA heuristically prunes reactions outside the cH set to generate the smallest network in which all cH reactions can carry a flux. A different method called integrative Metabolic Analysis Tool (iMAT) was proposed by Shlomi et al. [27]. iMAT maximizes the number of reactions whose flux is consistent with the measured gene-expression. First, a set of highly expressed reactions (rH) and a set of lowly expressed reactions (rL) are defined. Thereafter, iMAT finds a flux distribution that maximizes the sum of the number of rH reactions that carry a flux and the number of rL reactions that do not carry a flux. This flux distribution is predicted to be the part of the network that is active in the condition from which the gene expression measurements were taken.

Here we present a method that uses gene-expression as the sole source of information to build environment-specific metabolic models. We introduce a novel exploration of the alternative optimal solutions to the optimization problem proposed by Shlomi et al [27] and identify reactions that are active in all optimal solutions, which we call high-frequency reactions (HFR). Hypothesizing that HFR are likely to be active in the environment under study, we build environment-specific models by minimizing the size of the network subject to the constraint that all HFR should be able to carry flux. Using this method we modeled the metabolic states of *S. cerevisiae* growing on yeast-extract peptone media, supplemented with either glucose or ethanol. Our modeling predicts, with high sensitivity, genes that are essential for growth specifically on glucose or on ethanol. Remarkably, we predict the Crabtree effect [29] in yeast cells growing on glucose, and that cells growing on ethanol rely exclusively on oxidative phosphorylation for ATP synthesis. All these predictions, we emphasize, were achieved without knowledge of which nutrients are available to the cells. Importantly, such detailed and physiologically relevant predictions have not been achieved by any other method we are aware of.

## Results/Discussion

The study of metabolic fluxes is enormously aided by genome-scale reconstructions of metabolic networks. The large size of these networks, however, entails the challenge of identifying the actual metabolic state of living cells among a very large number of possibilities. Here we present a method that integrates gene-expression measurements with genome-scale models of metabolism to: i) identify reactions that are likely to be active, ii) constrain the space of possible flux distributions, and iii) predict the metabolic states of cells growing in completely uncharacterized environments. We applied this method to model the metabolism of *S. cerevisiae* growing on two different media, uncovering essential aspects of yeast energy metabolism.

### Metabolic constraint-based model

In this study we used genome-scale constraint-based model of yeast metabolism by Mo et al. [30] with modifications introduced by Szappanos et al. [31]. As we aimed at modeling metabolism in situations where nutrient availability is unknown, we enabled the free uptake or secretion of all exchange metabolites in the model. To obtain a gapless model, we identified dead-end reactions using Flux Variability Analysis (FVA) [32] and deleted them. The resulting gapless model, with unrestricted uptake or secretion of all exchange metabolites, was used as a starting point for our method. It comprised 777 metabolites, 710 genes and 1092 reactions, and is provided in the Supplementary Dataset S1.

### Classifying reactions as highly or lowly expressed

The first step into constraining the space of possible flux distributions using gene-expression measurements is to translate these measurements into reaction-expression calls. Gene expression measurements for yeast cultures growing on YPD (yeast-extract, peptone, dextrose) and YPEtOH (yeast-extract, peptone, ethanol) were obtained from Gasch et al [33]. For each condition, we ordered genes according to their expression level and classified the top 15% highest expressed genes as ‘highly expressed’ and the bottom 15% lowest expressed genes as ‘lowly expressed’ (Supplementary Table S1). Our choice of these 15% thresholds was motivated by the observation that the distribution of gene-expression values was approximately log-normal. Hence these threshold would classify genes with expression values about one standard deviation above and below the population's mean as highly or lowly expressed, respectively.

Gene-expression calls defined as described above were translated into reaction-expression calls using the metabolic model's detailed Boolean gene-to-reaction mapping ([31], Supplementary Table S2). The number of highly expressed reactions was similar in YPD and YPEtOH (194 and 184, respectively) whereas the number of lowly expressed reactions was lower in YPD (94) than in YPEtOH (144). The identities of reactions classified as highly (rH) or lowly expressed (rL) are reported in the Supplementary Table S3.

### Predicting active and inactive reactions

We formulated predictions of which reactions are likely to be active or inactive in YPD or YPEtOH by integrating gene-expression measurements with a model of yeast metabolism. First we used iMAT [27], which is described by Eqs. 1–6, to maximize the number of reactions whose flux is consistent with the measured gene-expression, the so-called agreement score. The inputs of this optimization are: a stoichiometry matrix, two vectors containing the upper and lower flux bounds for each reaction, and two sets of reactions classified by their expression as highly expressed (rH) or as lowly expressed (rL). Reactions that are neither highly nor lowly expressed do not influence the optimization, but many will be included in the resulting flux distribution. As an illustration, Figure 1 shows a toy metabolic network with the flux distribution obtained using iMAT highlighted in blue. Note that this flux distribution is consistent with expression measurements of four (r2, r3, r5 and r6) of the five reactions classified as either highly or lowly expressed. Further note that the optimal flux distribution includes reactions that are neither highly nor lowly expressed (i.e. r4, r8, r9 and r10).

**Figure 3:**
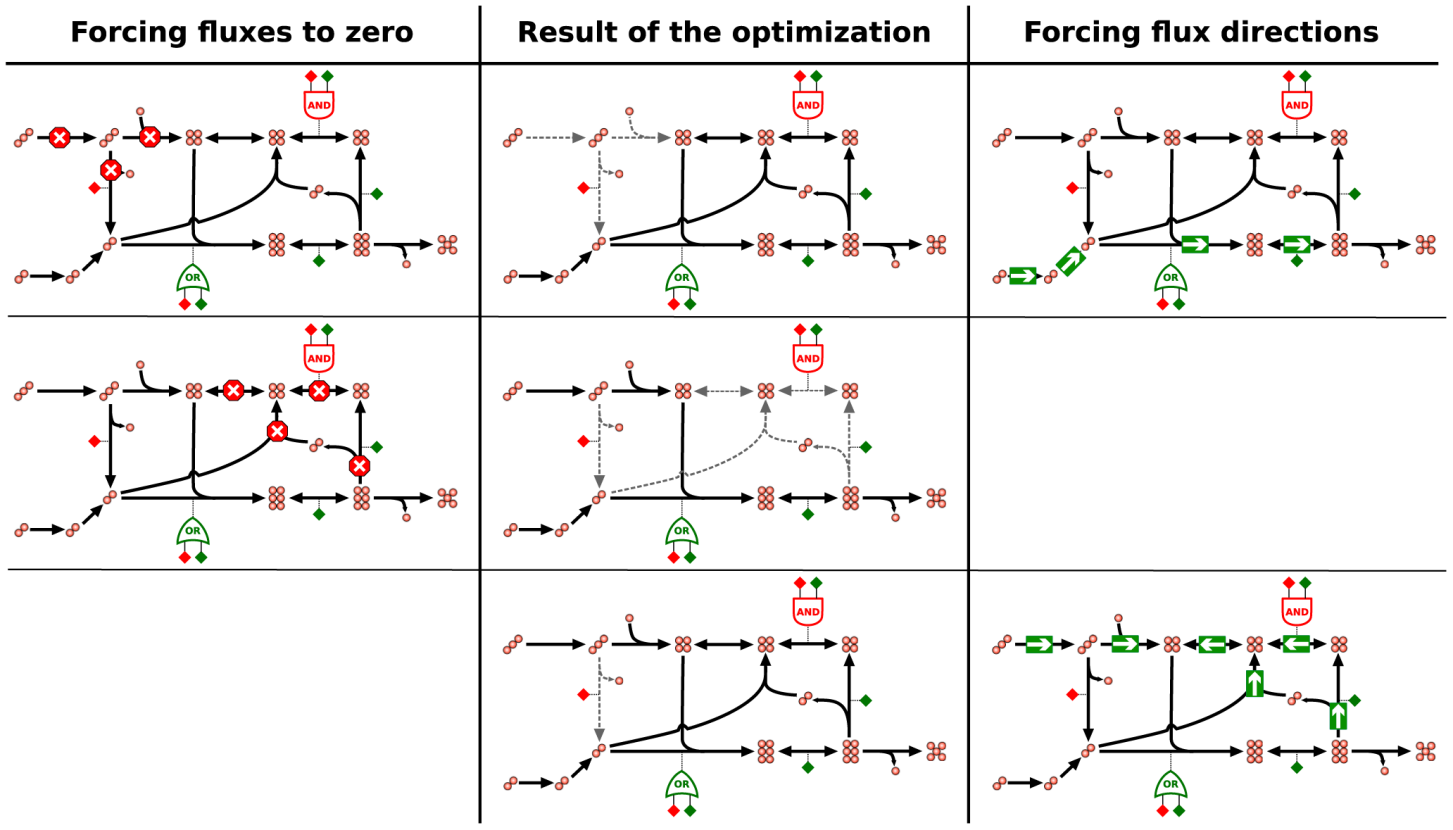
Exploration of alternative optimal flux distributions. Three alternative flux distributions that maximize the number of reactions whose flux is consistent with their gene expression, for the toy pathway in Figure 1, are shown in the central column. The panels to the left and right summarize the enzyme modulations that force the MILP solver to find the flux distributions in the central column. The modulations and resulting flux distributions are organized by rows. The leftmost column summarizes the reactions whose inactivation result in the finding of the flux distribution in the central column. Note that there are multiple reactions that when forced to be inactive each give rise to the same flux distribution in the central column. Note furthermore that the flux distribution in the third row cannot be found by the inactivation of a reaction. The rightmost column summarizes the reactions that when forced to carry a flux in the indicated directions, enable finding the flux distributions in the central column. Like with the inactivation of reactions, there are multiple reactions that when forced to carry flux in the indicated direction give rise to the same flux distribution. The flux distribution in the second row of the central column cannot be found by forcing any reaction to be active.

Our method to predict reactions that are likely to be active or inactive, stems from the realization that the flux distribution obtained by iMAT is one among many that maximize the agreement score. For example, in our toy model, three of the eight possible flux distributions maximize the agreement score (Figure 3). In order to circumvent the arbitrariness of the particular flux distribution returned by the Mixed Integer Linear Program (MILP) solver in a single optimization, we made the MILP solver explore the space of alternative optima by forcing each and every reaction in the network to be: i) inactive, ii) to proceed in the forward direction and, in the case of reversible reactions, iii) to proceed in the reverse direction as well. In each of these cases, we maximized the agreement score of the modified optimization problem, and selected those solutions achieving the maximum agreement score. For our toy model, Figure 3 summarizes the reaction modulations with which the three alternative flux distributions that maximize the agreement score are found. Note that different sets of reaction modulations force the MILP solver upon the same flux distribution, and that neither systematic inactivation of reactions nor systematic forcing of reactions to be active, separately, suffices to find all alternative optimal flux distributions. Notably, not all enzyme modulations yield optimization problems that can be solved, and among those that can be solved, some achieve suboptimal agreement scores.

As a means to predict which reactions are likely to be active or inactive in the condition of interest, we counted the number of times that each reaction was found to be active among the alternative optimal flux distributions. Reactions that were active in all alternative optimal solutions defined a set of high-frequency reactions (HFR) and are predicted to be active. Reactions that were always inactive defined a set of zero-frequency-reactions (ZFR) and are predicted to be inactive. The number of ZFR in YPD and YPEtOH (76 and 95, respectively) was lower than that of HFR (433 and 414, respectively). It is important to note that HFR and ZFR sets comprise different reactions than the rH and rL sets. For instance, not all highly expressed reactions are within the HFR set. Moreover, the HFR set includes some reactions that are lowly expressed (12 and 24 reactions for YPD and YPEtOH, respectively). The resulting sets of ZFR and HFR for yeast cultures growing on YPD or YPEtOH are reported in the Supplementary Table S4.

### Building environment-specific metabolic models

**Figure 4:**
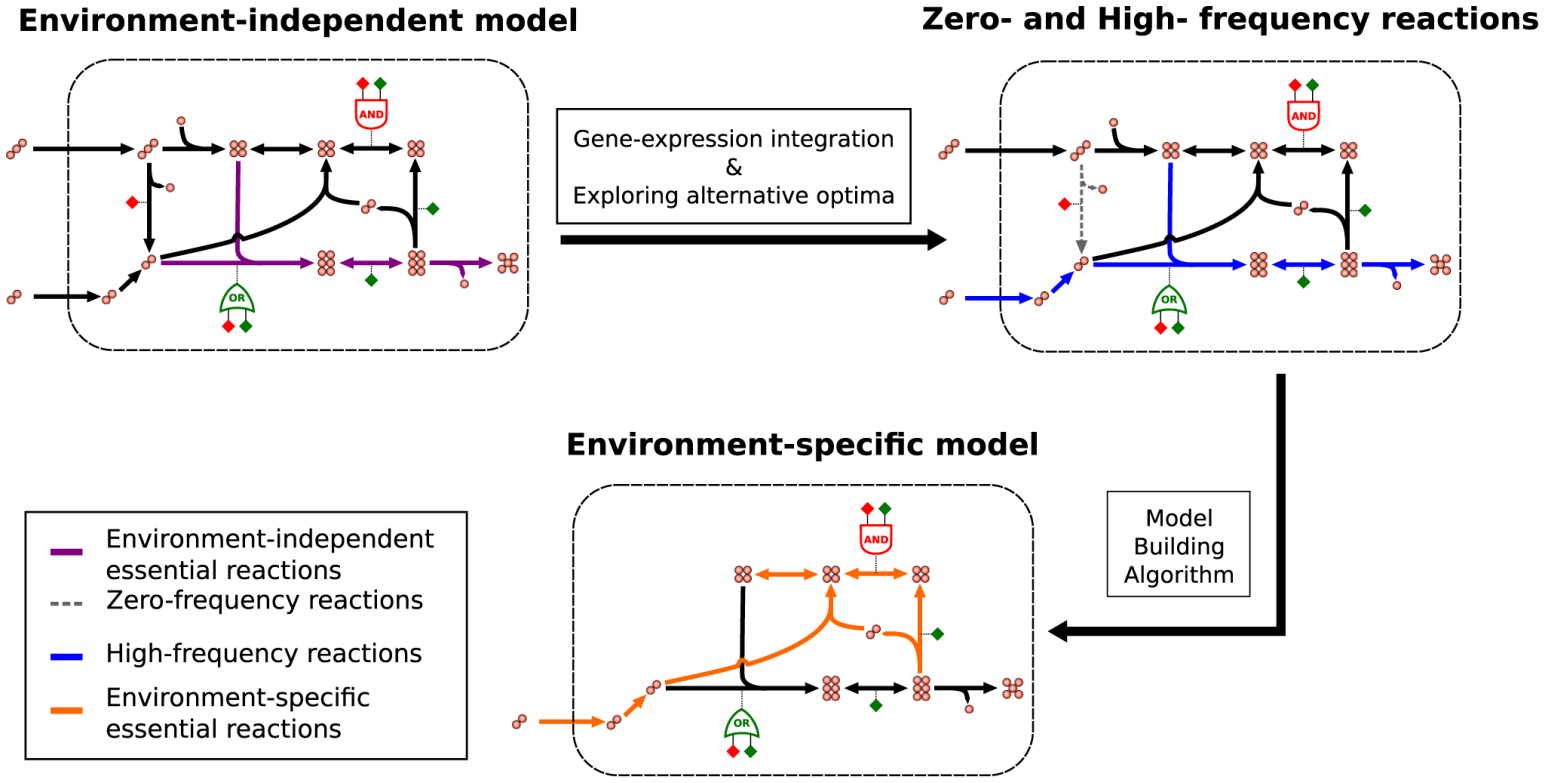
EXploration of Alternative Metabolic Optima (EXAMO). Environment-specific metabolic models are built in a two-step process. First, gene expression measurements are integrated with the original, environment-independent model. The exploration of alternative flux distributions that maximize the agreement score yields two sets of reactions: zero- and high-frequency reactions. Second, zero-frequency reactions are deleted and the Model Building Algorithm [26] is used to reduce the network with the constraint that all high-frequency reactions should be able to carry flux.

We built environment-specific metabolic models hypothesizing that HFR and ZFR are likely to be active and inactive, respectively, in the condition of interest. To this end we first deleted all ZFR, and thereafter used MBA to minimize the size of the network with the constraint that all HFR must be able to carry flux. We call our method ‘EXploration of Alternative Metabolic Optima’ (EXAMO), and summarize it in Figure 4. The YPD- and YPEtOH-specific models resulting from this procedure include about 50% of the reaction in the parent model. The overlap of the reactions in the YPD and YPEtOH models is 60%. Hence although similar in size, these two models are very different in content. The Supplementary Dataset S2 includes python scripts that detail and implement EXAMO, and step-by-step instructions for running these python scripts. The Supplementary Dataset S1 reports the resulting YPD- and YPEtOH-specific models as lists of reactions.

### Predicting environment-specific essential genes

Genome-wide measurements of essential genes have been used frequently as a means to validate constraint-based models of metabolism (e.g. [34]). Moreover, these models have been successful in predicting genes that are essential for the growth of pathogens, opening new avenues in the identification of drug targets to fight infection [6]. Frequently, the nutrients available to pathogens in host environments are largely unknown. In such situations, metabolic models are used to predict the set of genes that would be essential for growth in any environment. These environment-independent essential genes are predicted using Flux Balance Analysis (FBA) based gene essentiality analysis [34] in models where the free uptake or secretion of all exchange metabolites is enabled.

However, it is well known that nutrient availability determines the essentiality of genes [35]. For example, the *ICL1* gene in *S. cerevisiae*, is essential for growth on minimal medium when acetate or ethanol are provided as carbon sources, but is dispensable for growth on sugars or three-carbon substrates [36]. Building environment-specific models without knowing the environment but relying on gene-expression measurements can be seen as an effort to decode how the transcriptome describes the cells' environment. The extent to which this decoding is successful can be tested by contrasting the sensitivity of real cells to gene deletions with the predictions made with our environment-specific models.

To address this issue, we contrasted our model predictions with the results of two large-scale gene essentiality studies in *S. cerevisiae* [37], [38]. In the first study, by Giaever et al. [37], a library of single gene deletion mutants was constructed on YPD, covering 96% of the annotated open reading frames in *S. cerevisiae*. Single-gene deletion mutants that failed to grow on YPD were identified as essential for growth in this medium. The second study, by Snitkin et al [38], measured the growth phenotype of 465 of the single gene deletion mutants constructed by Giaever et al in 16 different conditions. Of these 16 conditions, we chose to model growth on YPEtOH because gene-expression measurements were available [33], and because it yielded one of the highest numbers of environment-specific essential genes [38].

Using FBA-based gene essentiality analysis [34], we computed lists of genes that are predicted to be essential for growth by the YPD and YPEtOH metabolic models we built with EXAMO. For YPD, the predicted essential genes could be compared directly with the measured growth phenotypes, but in the case of YPEtOH the scope of the measurements excludes genes that are essential for growth on YPD, since these mutants were unavailable for testing on YPEtOH. For this reason, the sets of genes measured to be essential for growth on YPD were subtracted from both, the set of genes measured and the set of genes predicted to be essential for growth on YPEtOH. The sensitivity of these predictions indicated that EXAMO uncovered 61% of the genes essential for growth on YPD and 77% of the genes essential for growth on YPEtOH. The positive predictive values of the predictions were 26% and 18% for YPD and YPEtOH, respectively.

**Figure 5:**
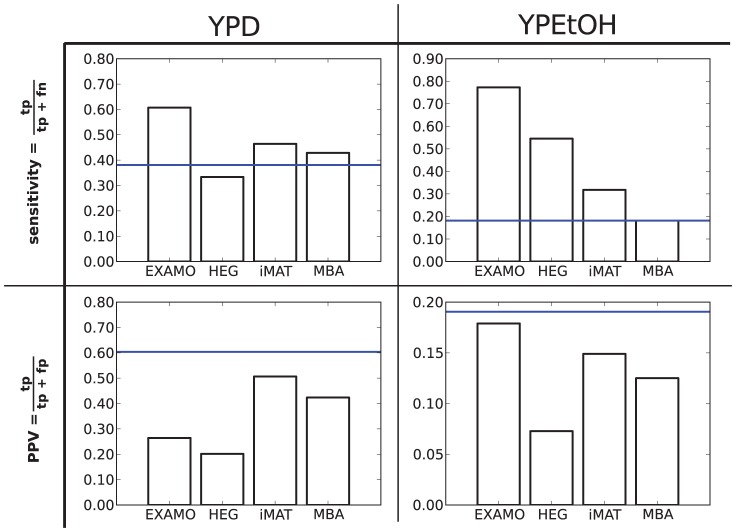
Comparing the quality of gene essentiality predictions. The sensitivities and positive predictive values (PPV) of the prediction of essential genes are shown for YPD and YPEtOH. The methods compared are EXploration of Alternative Metabolic Optima (EXAMO), Highly Expressed Genes as proxies for essential genes (HEG), Shlomi et al's integrative Metabolic Analysis Tool (iMAT, Eqs. 1–6) [27], and Jerby et al. 's Model Building Algorithm (MBA) [26]. The blue horizontal line marks the sensitivities and PPVs achieved with the yeast model when the free uptake and secretion of all exchange metabolites is allowed. These genes predicted to be essential for growth using the original yeast model, unconstrained by gene expression measurements, are predicted to be essential regardless of which nutrients are available to the cells.

Figure 5 compares the sensitivity and positive predictive value of our predictions (EXAMO) with those obtained with: i) high-expression as a proxy for essentiality (HEG), ii) FBA-based gene essentiality analysis using a model constructed with Shlomi et al.'s iMAT [27] (Eqs. 1–6), and iii) FBA-based gene essentiality analysis using a model constructed with Jerby et al.'s MBA [26]. For both YPD and YPEtOH, EXAMO achieves the highest sensitivities. The positive predictive value of all methods is below that of the environment-independent model (horizontal blue line), implying that the discovery of environment-specific essential genes is achieved at the cost of some false positive predictions. We evaluated the influence of the thresholds used to classify genes according to their expression levels by evaluating the sensitivities and positive predictive values using 10 and 20% as thresholds. For the thresholds tested, the sensitivity of the predictions made with EXAMO were the highest, whereas different methods performed best for different thresholds with regard to their positive predictive values (Supplementary Figure S1).

### Model predictions of metabolic states reveal aspects of yeast physiology

By building environment-specific models of metabolism, we predict drastically reduced spaces of possible flux distributions available to yeast cultures growing on YPD of YPEtOH. We then asked whether we can use these models to infer the actual metabolic state of these cells and, thereby, reveal aspects of yeast physiology. As a means of predicting a specific flux distribution, a point within the flux distribution space, we minimize the sum of all fluxes [39], [40]. Minimizing the sum of fluxes, rather than maximizing biomass production, has the advantage of not having to impose boundaries on the uptake of nutrients, and is thus compatible with the condition of not having information about nutrient availability. It implies the hypothesis that cells economize the amount of catalyst invested in metabolic networks. Here we propose a novel minimization of the sum of fluxes that is subject to the constraint that all HFR and the biomass synthesis reaction must carry flux (Eqs. 8–13).

Supplementary Tables S5 and S6 report the flux distributions we predict for yeast cells growing on YPD and YPEtOH, respectively. In this discussion we focus our attention on the predicted energy metabolism of these two cultures. For YPD cultures, our modeling predicts that 99% of the ATP production is ascribable to the glycolytic enzymes phosphoglycerate kinase and pyruvate kinase, that glucose (and not another sugar) serves as the cells' main energy source, and that 90% of the consumed glucose is fermented. Importantly, we also predict that oxidative phosphorylation is not impaired and that indeed it is responsible for a small fraction of the ATP production. These predictions are in large agreement with the repeatedly observed Crabtree effect [29], which describes the phenomenon whereby yeast ferments glucose aerobically in the presence of high glucose concentrations. It may be noted that the maximization of biomass production, the most commonly used objective function used in FBA studies, is at odds with the Crabtree effect, because fermentation of glucose leads to lower biomass yields than its respiration [41]. Contrary to our expectations, however, the regeneration of the 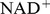 consumed by glyceraldehyde-3-phosphate dehydrogenase is not predicted to occur through the production of ethanol by alcohol dehydrogenase. Our modeling predicts that indole-3-acetaldehyde acts as an electron acceptor forming indole-3-ethanol at a rate that closely matches that of glyceraldehyde-3-phosphate dehydrogenase. We should remark that the prediction of the Crabtree effect is not an artifact introduced by the minimizing the sum of fluxes by itself. Minimizing the sum of fluxes in the original yeast model without constraining HFR to carry flux, results in a flux distribution in which 80% of the NADH produced in the cytosol is respired. Thus not the predominantly fermentative metabolism associated with the Crabtree effect.

**Figure 6:**
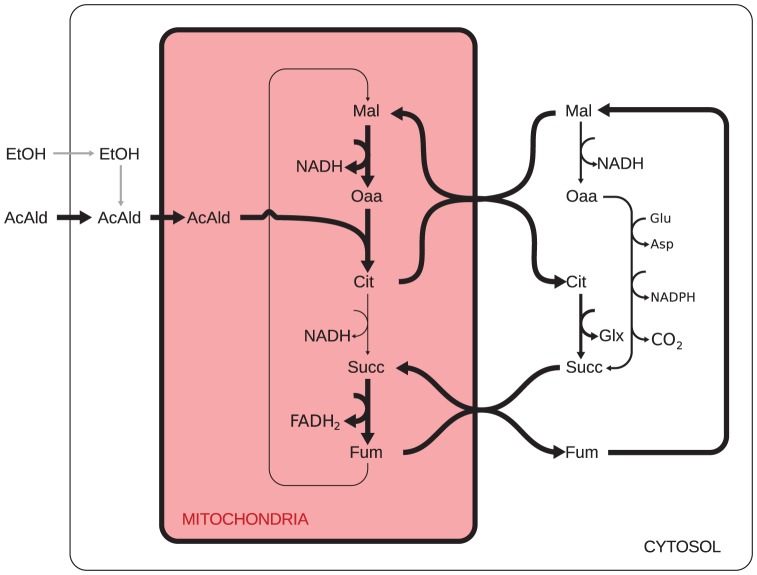
Predicted tricarboxylic acid cycle fluxes in YPEtOH cultures. The network of mitochondrial and cytosolic tricarboxylic acid cycle reactions is shown. The thickness of the arrows is drawn in proportion to the predicted fluxes. Acetaldehyde is predicted to be the main energy source and grey arrows indicate an alternative route through which ethanol could be used as energy source. In the cytosol a network of non-TCA reactions (glutamate decarboxylase, 4-aminobutyrate transaminase, succinate semialdehyde dehydrogenase, and aspartate transaminase) that convert oxaloacetate and glutamate into succinate and asparate with concomitant production of 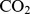 and NADPH is shown as a single arrow. Metabolite abbreviations: AcAld (acetaldehyde), Asp (aspartate), Cit (citrate), Fum (fumarate), Glu (glutamate), Glx (glyoxylate), Mal (malate), Oaa (oxaloacetate), Succ (succinate).

In stark contrast with our predictions for YPD cultures and in line with what is known about yeast physiology, for YPEtOH cultures we predict that *all* ATP is produced by oxidative phosphorylation. The main sources of electrons feeding the electron transport chain, and thus maintaining the proton motive force, are the reactions catalyzed by: aldehyde dehydrogenase (25%), malate dehydrogenase (26%) and succinate dehydrogenase (45%), with minor contributions by isocitrate dehydrogenase and pyruvate dehydrogenase. Our modeling predicts that acetaldehyde, which can be produced from ethanol via a single reaction, serves as the main energy source. Figure 6 shows the predicted fluxes through the TCA cycle. The majority (95%) of the succinate feeding the (mitochondrial) succinate dehydrogenase is produced outside the mitochondrion. About 60% of the cytosolic succinate is produced via isocitrate lyase, with the concomitant glyoxylate production being used to synthesize glycine. The remaining 40% is produced by a network of four reactions (glutamate decaboxylase, 4-aminobutyrate transaminase, succinate semialdehyde dehydrogenase, and aspartate transaminase), which overall stoichiometry involves the consumption equimolar amounts of oxaloacetate and glutamate to produce equimolar amounts of aspartate and succinate, with the concomitant production of NADPH and 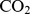. Our modeling predicts that the majority of NADPH (99%) is produced by the reaction catalyzed by succinate semialdehyde dehydrogenase, whereas the pentose phosphate pathway makes a negligible contribution. This latter prediction is supported by experiments in which yeast cells were grown in chemostats with ethanol as their only carbon source. These experiments suggest that indeed the contribution of the pentose phosphate pathway to the synthesis of NADPH is very minor [17]. The predictions achieved with EXAMO for YPEtOH are also beyond the capabilities of traditional FBA approaches, which in the absence of information about nutrient availability do not predict a two-carbon substrate as main energy source. Moreover, when the composition of YPEtOH is used to constrain the identity of metabolites that can be taken up, FBA predicts amino acids to serve as energy sources. Furthermore, it should be noted that neither iMAT nor MBA were capable of predicting the aspects of energy metabolism in YPD and YPEtOH described above.

In summary, we developed a method that used gene-expression measurements and genome-scale models of metabolism to infer the metabolic states of cells living in completely uncharacterized environments. Our method yields predictions about the essentiality of genes in specific environments with higher sensitivities than alternative methods. The metabolic states predicted by our method are in large agreement with what is known about yeast physiology with the prediction of the Crabtree effect as a prominent example. The consideration of the quality of gene-essentiality and metabolic state predictions together, encourages us to suggest that our method contributes to the decoding of the way the transcriptome represents the metabolic state of cells. Our method is also of immediate practical value, as it can be used to identify potential drug targets against infection that would be specifically effective within the host, where nutrient availability is largely unknown, or to distinguish metabolic states of health and diseased tissues *in vivo*. It may also be used to predict which nutrients are being consumed in complex industrial substrates, and contribute in research efforts towards the development of biofuel producing processes where many incompletely characterized media are tested.

## Methods

### Metabolic constraint-based model

We used the genome-scale constraint-based model of yeast metabolism by Mo et al. [30] with modifications introduced by Szappanos et al. [31]. This reconstruction comprises 1228 metabolites, 904 genes and 1575 reactions. With the objective of modeling metabolism in completely uncharacterized environments, we set the lower and upper boundaries of all exchange reactions to 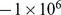 and 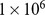, respectively. These changes enable the free uptake or secretion of all exchange metabolites in the model. Some of the model's reactions are dead-end reactions, incapable of carrying flux under any condition. We identified these reactions using FVA [32], and deleted them from the metabolic model. Metabolites that, after elimination of dead-end reactions, were not associated with any reaction were deleted as well. The resulting trimmed network comprised 777 metabolites, 710 genes and 1092 reactions.

### Classifying reactions as highly or lowly expressed

Our objective is to use gene-expression measurements to infer the metabolic state of cells growing in uncharacterized environments. To this end, we classified the reactions in the model as highly (rH) or as lowly (rL) expressed, based on gene-expression, using the constraint-based model's detailed Boolean gene-to-reaction mapping (Supplementary Table S2).

The model's Boolean gene-to-reaction mapping accounts for the fact that some enzymes are protein complexes, composed of proteins encoded by two or more genes, and for the existence of isoenzymes (cf. Figure 1). The requirement of several gene-products for one enzyme is described by the AND rule, whereas the possibility of different isoenzymes catalyzing the same reaction is described by the OR rule. For example, a reaction catalyzed by either one of two isoenzymes, each of which is a protein complex composed of two different gene products, would be described by the Boolean expression: 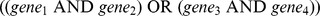. Where genes 1 and 2 constitute the first isoenzyme, and genes 3 and 4 constitute the second.

Gene-expression measurements for yeast growing on yeast-extract peptone medium supplemented with either glucose (YPD) or ethanol (YPEtOH), were taken from Gasch et al. [33]. We classified the top 15% highest expressed genes as ‘highly expressed’, and the bottom 15% lowest expressed genes as ‘lowly expressed’. The yeast model's gene-to-reaction mapping was then used to classify reactions based on the expression of their associated genes. The classification of reactions associated with single genes is straightforward. In the case of reactions catalyzed by protein complexes, the reaction is classified as lowly expressed if any of its associated genes is lowly expressed. It is classified as highly expressed if all of its associated genes are highly expressed. In the case of reactions catalyzed by isoenzymes, a reaction is classified as lowly expressed if all its isoforms are lowly expressed, and classified as highly expressed if any of the isoforms is highly expressed.

### Maximizing the agreement between fluxes and gene expression

The rH and rL reaction sets, composed on the basis of gene-expression measurements, were used as inputs of an optimization that searches for a flux distribution that maximizes the sum of two numbers: the number of rH reactions carrying flux, and the number of rL reactions that do not carry a flux. We will refer to this sum as the ‘agreement score’. This optimization was proposed by Shlomi et al. [27] and is subject to stoichiometric, thermodynamic and enzyme-capacity constraints. We added the constraint that the biomass reaction should have a non-zero flux. The optimization was formulated as a mixed integer linear program (MILP):
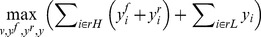
(1)

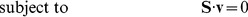
(2)

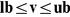
(3)

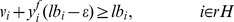
(4)

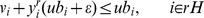
(5)

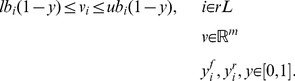
(6)The mass conservation constraint is enforced by Eq. 2, where **S** is an 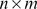 stoichiometric matrix, in which *n* is the number of metabolites and *m* is the number of reactions, and **v** is the flux vector. Thermodynamic (directional) and capacity constraints are enforced by Eq. 3, where **lb** and **ub** are the vectors containing lower and upper flux boundaries, respectively. For each reaction in the highly expressed set (rH), 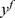 and 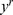 are Boolean variables. When 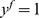, the flux through the reaction is forced to be larger than the threshold 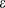 (Eq. 4). Conversely, when 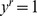, the flux is forced to be smaller than 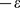, i.e. to proceed in the reverse direction (Eq. 5). Following Shlomi et al. [27] an 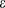 of 1.0 was used throughout this study. For each reaction in the lowly expressed set (rL), the Boolean variable *y* forces the flux through the reaction to zero when equal to 1 (Eq. 6).

### Exploring alternative optimal solutions

Solving the MILP in Eqs. 1 to 6 yields a flux distribution that maximizes the number of reactions whose fluxes are consistent with the expression of their genes. However, it is not the only possible flux distribution that achieves the maximum agreement score. In order to force the MILP solver to explore alternative optimal solutions, we modulated the flux through each reaction by changing its flux boundaries (Eq. 3). Each reaction was forced, in turn, to be inactive or to carry a positive flux (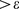). If the reaction was reversible, it was also forced to carry a negative flux (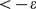), i.e. to proceed in the reverse direction (see Figure 3). For each reaction modulation we solved the MILP in Eqs. 1 to 6, and verified whether the maximum agreement score was achieved. In this way, we gathered a collection of flux distributions, all of which achieve the maximum agreement score. Next, we counted the number of times that each reaction was active among these alternative flux distributions, and defined two sets of reactions. High-frequency reactions (HFR) are those that are active in all alternative optima, and zero-frequency reactions (ZFR) are those that are inactive in all the alternative optima.

### Building environment-specific metabolic models for *S. cerevisiae*

Figure 4 summarizes the process of constructing environment-specific metabolic models. First, we defined a set of HFR and a set of ZFR by maximizing the agreement between fluxes and gene expression, and by exploring the alternative flux distributions that maximize the agreement score. Second, we deleted the set of ZFR and then used the MBA [26] to heuristically prune reactions outside the HFR set, with the constraint that all HFR should be able to carry flux. We will refer to this method for the construction of environment-specific models that starts with the exploration of alternative optima for the MILP in Eqs. 1–6, and that subsequently reduces the original model based on the ZFR and HFR, as ‘EXploration of Alternative Metabolic Optima’ (EXAMO), and provide an implementation of it in the Supplementary Dataset S2.

### Predicting environment-specific essential genes

As a means to validate these smaller environment-specific models we compared their gene-essentiality predictions with those of two large-scale gene essentiality studies of yeast growing on YPD or YPEtOH. Giaever et al. [37] constructed a library of single gene deletion mutants in YPD, covering 96% of the annotated open reading frames in *S. cerevisiae*. Snitkin et al [38] tested 465 of these YPD-viable mutants for growth on YPEtOH.

We used our environment-specific metabolic models to predict essential genes using FBA [34]. We then calculated the sensitivity (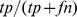) and the positive predictive value (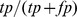) of our predictions. Where true positive predictions (*tp*) refer to correctly predicted essential genes, false negatives (*fn*) refer to genes predicted to be dispensable that are actually essential, and false positives (*fp*) refer to genes predicted to be essential but that are actually dispensable. Because the mutant library was constructed in YPD, mutants that would be essential for growth in YPD but dispensable for growth in some other medium are not available. For this reason, the scope of the gene essentiality study on YPEtOH excludes genes that are essential for growth in YPD. Thus, genes that are essential for growth on YPD were excluded in the prediction sensitivity and positive predictive value of YPEtOH.

To further appraise the usefulness of our method, we compared its predictions to those of two other methods that integrate gene-expression with constraint-based models of metabolism: Jerby et al. 's MBA [26] and Shlomi et al. 's iMAT (Eqs. 1–6) [27]. Following Frezza et al. [28], for MBA we chose to use the rH as the high-probability reaction set. For iMAT, we used the same rH and rL sets as we used for EXAMO. We also compared our predictions with those generated using highly expressed genes as predictors for essentiality. Highly expressed genes were defined as the 15% highest expressed genes. The quality parameters: sensitivity and positive predictive value where calculated in the same was as for EXAMO.

### Predicting metabolic states

We hypothesized that HFR are likely to be active in the condition of interest. To investigate whether this hypothesis yields meaningful predictions for the metabolic state of the cells, we computed flux distributions for cells growing on YPD or YPEtOH by minimizing the sum of all fluxes in the environment-specific models subject to the constraint that HFR must carry flux. Minimizing the sum of fluxes requires that each reversible reaction is separated into a forward (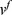) and a reverse (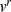) reaction, for irreversible reactions 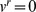. To minimize the sum of fluxes with the constraint that all HFR should carry flux and considering the fact that some HFRs are reversible, we wrote the following mixed integer linear program:
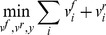
(7)

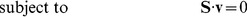
(8)

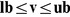
(9)

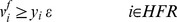
(10)

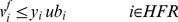
(11)

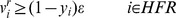
(12)

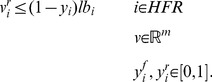
(13)The mass conservation constraint is enforced by Eq. 8, where **S** is a 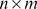 stoichiometric matrix, in which *n* is the number of metabolites and *m* is the number of reactions, and **v** is the flux vector. Thermodynamic (directional) and capacity constraints are enforced by Eq. 9, where **lb** and **ub** are the vectors containing lower and upper flux boundaries, respectively. For each reaction in the HFR set we defined an integer variable *y*. If the 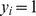, 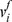 is bound to the interval 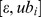 and 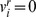, hence forcing the reaction to be active in the forward direction. On the other hand, if 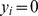, 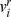 is bound to the interval 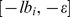 and 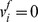, this forcing the reaction to be active in the reverse direction. In this study we chose a value of 1 for 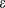 in all our calculations. Notice that these integer variables force reversible reactions to be active in either direction, prohibiting that the forward and reverse directions are simultaneously active. An implementation of the mixed integer linear program described above is provided in the Supplementary Dataset S2.

## Supporting Information

Dataset S1 **Constraint-based models as lists of reactions.** Versions of Mo et al's yeast metabolic model [30], are included as list of reactions in comma-separated-value (csv) files. All model files start with iMM904, the identifier used by Mo et al ([30] MM: Monica Mo, 904 genes). ‘deadEndRxnsDeleted’ identifies the model after deletion of dead-end reactions (see main text). ‘examo_glc’ and ‘examo_eth’ identify the YPD- and YPEtOH-specific models built using EXAMO. The ‘15’ that ends these two last file-names indicates the percentage threshold used to classify genes as highly or lowly expressed.
(ZIP)

Dataset S2 **EXAMO scripts.** Scripts for running the EXAMO software are provided. The user's manual lists the necessary dependencies and explains how to run the scripts and what input and output files are used and generated by each script.
(ZIP)

Figure S1 **Gene essentiality predictions varying gene expression thresholds.** The sensitivities and positive predictive values (PPV) of the predictions of essential genes are shown for YPD and YPEtOH for three different thresholds used to define highly and lowly expressed genes (see main text). The methods compared are: EXploration of Alternative Metabolic Optima (EXAMO –red circles), Shlomi et al's integrative Metabolic Analysis Tool (iMAT –blue diamonds) [27], and Jerby et al. 's Model Building Algorithm (MBA –green squares) [26]. The horizontal line marks the sensitivities and PPVs achieved with the yeast model when the free uptake and secretion of all exchange metabolites is allowed. These genes predicted to be essential for growth using the original yeast model, unconstrained by gene expression measurements, are predicted to be essential regardless of which nutrients are available to the cells.
(EPS)

Table S1 **Gene-expression calls.** Based on the gene expression measurements from yeast cells growing on glucose (YPD) or ethanol (YPEtOH) [33], we classified the top 15% highest expressed genes as ‘highly expressed’ (denoted with a ‘1’) and the bottom 15% lowest expressed genes as ‘lowly expressed’ (denoted with ‘−1’). Genes outside the highly and lowly expressed sets are indicated with a ‘0’.
(XLSX)

Table S2 **Boolean gene-to-reaction mapping.** The genome-scale constraint-based model of yeast metabolism by Mo et al [30] includes a detailed Boolean gene-to-reaction mapping, which is provided here as a list of reaction names with their associated reaction identifies (id) and their associated genes.
(XLSX)

Table S3 **Reaction-expression calls.** Reactions were classified by expression based on gene-expression calls (Supplementary Table S1) and the metabolic model's detailed Boolean gene-to-reaction mapping (Supplementary Table S2). This list of reaction includes only reactions that have one or more genes associated with them. Highly expressed reactions (rH) are indicated with a ‘1’, lowly expressed reactions with a ‘−1’ and all others with a ‘0’.
(XLSX)

Table S4 **Lists of high- and zero-frequency reactions for YPD and YPEtOH.** High- and zero-frequency reactions computed by integrating gene expression from yeast cultures grown on YPD or YPEtOH [33] and a genome-scale model of yeast metabolism [30]. High-frequency reactions (HFR) are those that are always active among the all alternative optimal flux distributions and zero-frequency reactions (ZFR) are those that are always inactive (see main text).
(XLSX)

Table S5 **Metabolic state of yeast cells growing on YPD.** Reaction stoichiometries, identifiers, names, associated genes and predicted fluxes are listed. Reactions with absolute fluxes lower than 10E-10 are not shown.
(XLSX)

Table S6 **Metabolic state of yeast cells growing on YPEtOH.** Reaction stoichiometries, identifiers, names, associated genes and predicted fluxes are listed. Reactions with absolute fluxes lower than 10E-10 are not shown.
(XLSX)
