# Supplementary material for: Inferring Metabolic States in Uncharacterized Environments Using Gene-Expression Measurements
Source: PLoS Comput Biol. 2013 Mar 21;9(3):e1002988. doi: 10.1371/journal.pcbi.1002988 (PMC3605102; doi:10.1371/journal.pcbi.1002988)
Supplement: Dataset S2 — EXAMO scripts. Scripts for running the EXAMO software are provided. The user's manual lists the necessary dependencies and explains how to run the scripts and what input and output files are used and generated by each script. (ZIP) [file pcbi.1002988.s002.zip › dataset_S2/EXAMO_usersManual.pdf]

EXploration of Alternative Metabolic Optima (EXAMO)

User's manual

S. Rossell, M. A. Huynen & R. A. Notebaart

1 Dependencies

This implementation of EXAMO has the following dependencies:

- Python (tested with 2.6) (`http://www.python.org`)
- Numpy (`http://numpy.scipy.org`)
- Scipy (`http://www.scipy.org`)
- Gurobi and its python interface (`http://www.gurobi.com`)

2 Inputs

EXAMO requires as inputs: i) a gapless reconstruction of a metabolic network and ii) a table of genes classified by their expression.

2.1 Metabolic reconstruction

A python dictionary is used as input for EXAMO. This dictionary contains the ingredients of a constraint based model of metabolism. The dictionary is saved as a pickle file (`http://docs.python.org/2/library/pickle.html`) in the 'data/' directory. It comprises the following keys and values:

Key	Value Type	Value description
'S'	[scipy.sparse coo_matrix]	stoichiometry matrix
'idSp'	[list of strings]	list of ids of the model's metabolites
'idRs'	[list of strings]	list of ids of the model's reactions
'lb'	[list of floats]	lower flux boundaries for the model's reactions
'ub'	[list of floats]	upper flux boundaries for the model's reactions
'gene2rxn'	[dict]	gene-to-reaction mapping
'rxns'	[dict]	dictionary with the elements of each reaction
'genes'	[list of strings]	list of all genes in the model

As an example, below the ‘rxns’ dictionary for phosphofructokinase (PFK) i.e.`rxns['R_PFK']`:

```
{'genes': '( YGR240C  and  YMR205C )',
 'id': 'R_PFK',
 'index': 1246,
 'lb': 0.0,
 'metaid': '',
 'name': 'R_phosphofructokinase',
 'products': {'M_adp_c': 1.0, 'M_fdp_c': 1.0, 'M_h_c': 1.0},
 'reactants': {'M_atp_c': 1.0, 'M_f6p_c': 1.0},
 'reversible': False,
 'subsystem': 'S_GlycolysisGluconeogenesis',
 'ub': 999999.0}
```

2.2 Gene expression calls

Gene expression calls classify genes by their expression. For them to be read by the EXAMO scripts, they should be saved as comma-separated-value (csv) tables, and placed in the subdirectory ‘data/’.

Gene Symbol	gene call
YAL028AW	0
YAL028W	0
YAL029C	1
YAL030W	0
YAL031C	0
YAL032C	-1
YAL033W	1
YAL034AW	-1
YAL034C	0
YAL034W-A	0
YAL035C-A	1
YAL035W	0
YAL036C	0
YAL037W	0
⋮	⋮

Where the gene symbol must correspond to the gene symbol used in the metabolic reconstructions. Highly expressed genes are associated with a gene call of 1, and lowly expressed genes with a gene call of -1. All other genes, with intermediate expression are associated with a gene call of 0.

3 Running the scripts

To build EXAMO environment-specific models, the following scripts should be run in the order listed.

1. `_01_findZeroAndHighFreqRxns.py`
2. `_02_minimizeNetwork_part_A.py`
3. `_03_minimizeNetwork_part_B.py`
4. `_04_predictMetabolicState.py`

Each script includes a brief description of its inputs and outputs. There is an input section in each script that the user can modify to fit his or her needs. In call cases, the scripts are executed from the comand line by simply typing:

```
python nameOfTheScript.py
```

3.1 _01_findZeroAndHighFreqRxns.py

This first script accepts comma-separated-value table of genes classified by expression. It should be placed in the 'data/' directory and should be named geneCalls_[*description*].csv. The gene-expression calls used in the manuscript are included in the 'data/' directory. One for yeast cells grown on glucose (`geneCalls_glc_15_85.csv`), and another for yeast cells grown on ethanol (`geneCalls_eth_15_85.csv`). Notice that `glc_15_85` and `eth_15_85` would be the descriptions for glucose and ethanol, respectively.

The second input is a dictionary containing the constraint-based model. The 'data/' directory includes the dictionary `iMM904_blkRxnsDeleted_dict.pkl`, in which all dead end, blocked reactions, have been removed.

The outputs of this script are saved to the 'data/' directory using python's pickle package. The first output file: rxnsClassifiedByExpression_[*description*].pkl is a dictionary with keys: 'rL', 'rU', and 'rH', which values are sets of reactions classified as lowly, intermediately, and highly expressed, respectively. The classification of these reactions is achieved using gene calls as inputs and the model's detailed gene-to-reaction mapping.

The second output file: freqBasedRxns_[*description*].pkl is a dictionary with keys 'zfr' and 'hfr', which values are the sets of zero-frequency and high-frequency reactions that result from the exploration of alternative optima to the maximization of the agreement between fluxes and gene expression.

3.2 _02_minimizeNetwork_part_A.py

This script performs the first part of Jerby et al's Model Building Algorithm (MBA) [1]. The user needs to specify the 'description' tag that was used in the first script, and the

script will import the corresponding zero- and high-frequency reaction sets. MBA is computationally very expensive, but its also an embarrassingly parallel problem. According to the computational power available, the user can specify 'numProc' to decide the number of parrallel process to be initiated, and 'numRep', the number of times the algorithm should be repeated. For each complete heuristic pruning, the script saves the list of reactions of each candidate model in a directory called 'data/mbaCandRxns' using python's pickle.

3.3 _03_minimizeNetwork_part_B.py

This script should be executed after _02_minimizeNetwork_part_A.py has been completed. It accepts the description tag chosen in the first script. It reads all the candidate reaction lists in the 'data/mbaCandRxns' that have the description tag and prints the average overlap between the candidate reaction sets, and its standard deviation. The number of times that a reaction occurs among all candidate reaction lists is used to classify reactions into sets of descending order in the frequency with which they are present in all candidate reaction lists. Starting with the set of reactions most frequently encountered in all candidate reaction lists and proceeding in descending order, reaction sets are added to the high-frequency reaction set until a network in which all HFR can carry flux is obtained (cf. [1]). This resulting network is the environment-specific metabolic network we aimed to construct. The environment-specific model obtained is saved as a python dictionary using python's pickle. It comprises the same keys as the metabolic model used as input for _01_findZeroAndHighFreqRxns.py, but includes less reactions, metabolites, and genes.

3.4 _04_predictMetabolicState.py

Once an environment-specific model has been constructed, it is possible to ask whether these models can be used to infer the actual metabolic state of the cells from which the gene-expression measurements were taken. We propose to minimize the sum of fluxes [2, 3] as a means of predicting a specific flux distribution, a point within the flux distribution space. This script implements a novel minimization of the sum of fluxes that is subject to the constraint that all HFR and the biomass synthesis reaction must carry flux. The user needs only to specify the description tag chosen in the first script and execute the script. The output of the script is a comma-separated-value table listing the ids of the reactions with non-zero fluxes along with their corresponding flux value prediction.

4 A note on opening input and output files

All input and output files are either comma-separated-value tables (.csv) or python's pickle files (.pkl). The former, csv, can be opened in a spreadsheet program like Excel or Gnumeric, or as text. The latter, pkl, must be imported using python's pickle.

5 Summary of files in the 'data/' directory

- `iMM904_blkRxnsDeleted_dict.pkl`. Yeast metabolic model by Mo et al [4], modified by [5], with all dead-end reactions deleted.
- `iMM904_examo_glc_15_85_dict.pkl`. YPD specific model constructed with EXAMO using gene-expression measurements from [6].
- `iMM904_examo_glc_15_85_dict.pkl`. YPEtOH specific model constructed with EXAMO using gene-expression measurements from [6].
- `geneCalls_glc_15_85.csv`. YPD genes classified as highly (top 15%) or lowly (bottom 15%) expressed. Highly expressed genes are indicated with a 1, lowly expressed genes with a -1. All other genes are indicated with a 0.
- `geneCalls_eth_15_85.csv`. YPEtOH genes classified as highly (top 15%) or lowly (bottom 15%) expressed. Highly expressed genes are indicated with a 1, lowly expressed genes with a -1. All other genes are indicated with a 0.
- `rxnsClassifiedByExprssion_glc_15_85.pkl`. Reactions classified as highly (rH), lowly (rL) of intermediately (rU) expressed, for cultures grown on YPD.
- `rxnsClassifiedByExprssion_eth_15_85.pkl`. Reactions classified as highly (rH), lowly (rL) of intermediately (rU) expressed, for cultures grown on YPEtOH.
- `freqBasedRxns_glc_15_85.pkl`. High- and zero- frequency reactions for cultures grown on YPD.
- `freqBasedRxns_eth_15_85.pkl`. High- and zero- frequency reactions for cultures grown on YPEtOH.
- `metabolicState_glc_15_85.csv`. Predicted metabolic state for cells grown on YPD.
- `metabolicState_eth_15_85.csv`. Predicted metabolic state for cells grown on YPEtOH.

References

[1] Jerby L, Shlomi T, Ruppin E (2010) Cmputational reconstruction of tissue-specific metabolic models: application to human liver metabolism. Mol Syst Biol 6: 401.

[2] Holzhütter H (2004) The principle of flux minimization and its application to estimate stationary fluxes in metabolic networks. Eur J Biochem 271: 2905–2922.

[3] Schuetz R, Kuepfer L, Sauer U (2007) Systematic evaluation of objective functions for predicting intracellular fluxes in escherichia coli. Mol Syst Biol 3.

[4] Mo ML, Palsson BO, Herrgard MJ (2009) Connecting extracellular metabolomic measurements to intracellular flux states in yeast. BMC Syst Biol 3: 37.

[5] Szappanos B, Kovacs K, Szamecz B, Honti F, Costanzo M, et al. (2011) An integrated approach to characterize genetic interaction networks in yeast metabolism. Nat Genet 43: 656–662.

[6] Gasch AP, Spellman PT, Kao CM, Carmel-Harel O, Eisen MB, et al. (2000) Genomic expression programs in the response of yeast cells to environmental changes. Mol Biol Cell 11: 4241–4257.
